# The Diverse Roles of the Mucin Gene Cluster Located on Chromosome 11p15.5 in Colorectal Cancer

**DOI:** 10.3389/fcell.2020.00514

**Published:** 2020-06-30

**Authors:** Guo-Lian Gan, Jing Liu, Wen-Jia Chen, Qian-Qian Ye, Ya Xu, Hua-Tao Wu, Wei Li

**Affiliations:** ^1^Department of General Surgery, The First Affiliated Hospital of Shantou University Medical College, Shantou, China; ^2^Changjiang Scholar’s Laboratory/Guangdong Provincial Key Laboratory for Diagnosis and Treatment of Breast Cancer, Shantou University Medical College, Shantou, China; ^3^Department of Physiology/Cancer Research Center, Shantou University Medical College, Shantou, China

**Keywords:** mucin, colorectal cancer, dysfunction, occurrence, cell adhesion

## Abstract

Colorectal cancer (CRC), the third most common malignant tumor in the world, shows multiple complex and pathologies based on the impaired structure and function of the intestinal mucosal barrier. Goblet cells secrete mucins, which are involved in the formation of the intestinal mucosal barrier and not only lubricate and protect the intestinal mucosa but also participate in the processes of cell adhesion, intercellular signal transduction, and immune regulation. It is accepted that the disordered expression and dysfunction of mucins are associated with the occurrence and development of CRC. This article focuses on the secretory mucins encoded by a gene cluster located on chromosome 11p15.5 and systematically reviews their composition, regulation, function, and role in CRC, to deepen the understanding of the pathogeneses of CRC and to provide a new basis and ideas for the treatment of CRC.

## Introduction

Globally, the incidence of colorectal cancers (CRCs) ranks third among common malignant tumors, and CRC has the second highest mortality (Bray et al., 2018). Even considering the lifestyle and diet habits of different ethnicities, the incidence and mortality of CRCs are still increasing year by year (Global Burden of Disease Cancer Collaboration et al., 2017). In China, 300,000 individuals are newly diagnosed with CRC annually, with an average 4.2% increase yearly (Chen et al., 2018). The incidence of CRC in males is higher than that in females, and urban areas have a higher CRC incidence than rural regions (Chen et al., 2018), suggesting that the etiological mechanism of CRC is complex and multifactorial as the result of the combined effects of genetic and environmental factors.

It is accepted that the normal function of the intestinal mucosal barrier is important for maintaining digestion and absorption and preventing abnormal disease, while rupture and dysfunction of the intestinal mucosal barrier can lead to a series of pathophysiological changes in the intestinal mucosa and eventually cause the occurrence of malignant tumors and CRCs (Yu, 2018). On the intestinal mucosal barrier, the gelatinous mucus layer covers the mucosal surface and functions as the “first line” to lubricate and protect the intestinal mucosa (Korytowski et al., 2017). More importantly, this layer also participates in the processes of intercellular adhesion, signal transduction, and immune regulation (Jakiela et al., 2014).

Among the components of the gelatinous mucus layer, mucins (MUCs), mainly secreted by goblet cells (GCs), are a family of highly glycosylated proteins with high molecular weights that are widely distributed in various tissues and organs (O’Connell et al., 2002). To date, 27 MUC proteins have been identified, and they are divided into secretory and membrane-bound types, based on their forms (Table 1). Membrane-bound mucins (MUC1, MUC3A, MUC3B, MUC4, MUC12, MUC13, MUC15, MUC16, MUC17, MUC20, and MUC21) exhibit hydrophobic sequences or “transmembrane domains” responsible for anchoring them in the lipid bilayer and have C-terminal peptides that enter the cytosol. The secretory mucins (MUC2, MUC5AC, MUC5B, MUC6, MUC8, and MUC19) with one exception (MUC7) possess one or several von Willebrand factor (vWF)-like D domains, and cysteine-rich peptides, which function in the oligomerization of mucin monomers and in packaging into secretory vesicles (Lehmann et al., 1989; Perez-Vilar and Hill, 1999; Moniaux et al., 2001; Chen et al., 2004; Higuchi et al., 2004; Itoh et al., 2008; Chairatana and Nolan, 2017). According to whether they are capable of forming a gel, the secretory mucins can be further divided into two subtypes, gel-forming and soluble MUCs. Interestingly, MUC2, MUC5AC, MUC5B, and MUC6 belong to gel-forming MUCs and are located near each other on human chromosome 11p15.5 (Pigny et al., 1996a). Unsurprisingly, gel-forming MUCs have similar regulation pathways and functions in the normal intestinal mucosa (Walsh et al., 2013). However, their expression has tissue and cell specificity, and abnormal levels of these MUCs have been found in many diseases, especially CRCs. Hence, this article focused on the secretory type of mucins, MUC2, MUC5AC, MUC5B, and MUC6, located on chromosome 11p15.5, to systematically review their composition, regulation, function, and role in CRC, to deepen the understanding of the pathogeneses of CRC and provide a new basis and ideas for the treatment strategies of CRC.

**TABLE 1:** The classification of human mucin gene family.

**Gene symbol**	**Cytogenetic band**	**HGNC ID (gene)**	**Length (aa)**	**Molecular weight (kDa)**	Unique_idf*
**Secreted mucins (gel-forming)**					
MUC2	11p15.5	7512	5,179	540.3	Q02817
MUC5AC	11p15.5	7515	5,654	585.6	P98088
MUC5B	11p15.5	7516	153	1602	H0YDX8
MUC6	11p15.5	7517	2,439	257	Q6W4X9
**Secreted mucins (soluble)**					
MUC7	4q13.3	7518	377	39.2	Q8TAX7
MUC8	12q24.33	7519	313	33.3	Q12964
MUC9	1p13.2	8524	678	75.4	Q12889
MUC19	12q12	14362	8,384	805.3	Q7Z5P9
**Membrane-associated mucins**					
MUC1	1q22	7508	1,255	122.1	P15941
MUC3A	7q22.1	7513	3,323	345.1	Q02505
MUC3B	7q22	13384	1,237	131.4	Q9H195
MUC4	3q29	7514	1,176	130.4	Q99102
MUC12	7q22.1	7510	5,478	558.2	Q9UKN1
MUC13	3q21.2	7511	512	54.6	Q9H3R2
MUC14	4q24	16041	261	27.5	Q9ULC0
MUC15	11p14.3	14956	334	36.3	Q8N387
MUC16	19p13.2	15582	14,507	1519.2	Q8WXI7
MUC17	7q22.1	16800	4,493	451.7	Q685J3
MUC20	3q29	23282	674	68.3	Q8N307
MUC21	6p21.33	21661	566	54.2	Q5SSG8
MUC22	6p21.33	39755	1,773	173.5	E2RYF6

*Unique and stable entry identifier. https://www.uniprot.org/.

## MUC2

**FIGURE 1:**
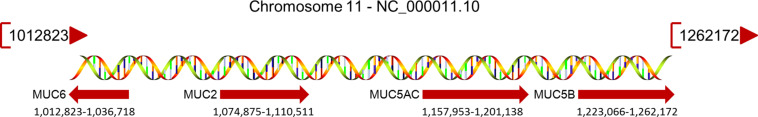
MUCs located on chromosome 11p15.5 and their relative positions. The MUC gene cluster spans a region of approximately 400 kb; the map extends to 770 kb, and the beginning and end of each gene are specified under their red arrow.

The MUC2 protein, encoded by the MUC2 gene (Figure 1), is the most abundant secreted MUC, covering the mucosal surface of the intestinal cavity in the form of gelatin and forming the basis of the mucous layer (Yamashita and Melo, 2018). The central domain of the MUC2 protein contains a large number of repetitive tandem threonine, serine, and proline polypeptides. The N-terminal domain of MUC2 contains the vWF D-like domain, rich in cysteine, while the C-terminal domain is also rich in cysteine (Rousseau et al., 2004).

**FIGURE 2:**
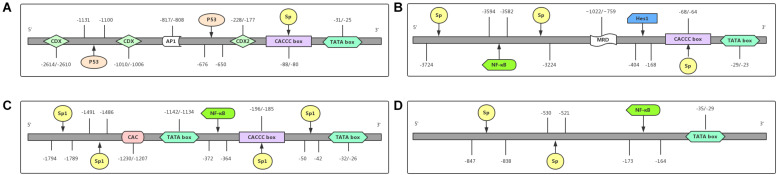
Promoter regions of MUCs and their functional domains. The promoter region of each gene contains different binding sites for transcriptional factors. **(A)** MUC2 is regulated at the transcriptional level by the transcriptional factors CDX, CDX2, P53, and Sp. **(B)** MUC5AC is transcriptionally regulated by the transcriptional factors NF-κB, Hes1, and Sp. **(C)** The transcriptional level of MUC5B is regulated by the transcriptional factors NF-κB and Sp1. **(D)** MUC6 is regulated at the transcriptional level by the transcriptional factors NF-κB and Sp. Abbreviations: CDX (caudal-related homeobox); Sp. (signal peptide); AP1 (activator protein 1); NF-κB (nuclear factor-κB); P53 (a tumor suppressor gene, binding to p53-responsive elements in the promoter region); Hes1 (hes family bHLH transcription factor 1).

As shown in Figure 2A, the promoter region of the MUC2 gene includes TATA-binding sites, the CCAAT box, and many other transcription factors binding sites, predicting its multiple transcriptional regulatory pathways, and these features result in the specific expression of the MUC2 gene in goblet cells (Fagerberg et al., 2014). Velcich et al. (1997) isolated a group of overlapping codons harboring the entire MUC2 locus, and found maximal transcriptional activity in the promoter region of the MUC2 gene, from the AUG translational initiation codon +1 to −848, in several intestinal cell lines, and this area did not promote the expression of MUC2 in HeLa cervical cancer cells. Many transcription factors can regulate the expression of the MUC2 gene by binding with its promoter of the MUC2 gene. As SRY-box transcription factor 9 (SOX9) is a high-mobility group box transcription factor, Blache et al. (2004) found that SOX9, downstream of Wnt signaling pathway can transcriptionally inhibit the MUC2 gene. The tumor suppressor p53 directly binds to the promoter of the MUC2 gene and transcriptionally induces the expression of the MUC2 gene in response to cell stress in human colon cancer cells, as well as in breast cancer cells (Ookawa et al., 2002). In colon cancer cells, galectin-3 modulated the expression of the MUC2 gene by forming a complex with AP-1 and directly binding to 1,500–2,186 bp region upstream of its translation start site (Song et al., 2005). The hormone somatostatin (SST) was also found to be involved in the regulation of the MUC2 gene to protect the colon. Song et al. (2019) reported that exogenous SST administration significantly increased colonic expression of the MUC2 gene and mucus secretion through the Notch-Hes1 pathway, while knockdown of SST receptor 5 (SSTR5) in human goblet cell-like LS174T cells effectively blocked the SST-induced increase in MUC2 gene expression and mucus secretion, indicating the role of the SST-SSTR5-Notch-Hes1-MUC2 axis in the regulation of colonic mucus formation.

As a secretory protein, the function of MUC2 is not only regulated by its expression, but also influenced by its exocytosis from colon goblet cells. McCool et al. (1995) found that LS180 colonic tumor cells could synthesize and secrete MUC2 without the addition of secretagogues; however, the disrupted Golgi and inhibition of microtubule assembly inhibited baseline secretion of MUC2, and actin microfilaments was involved in regulated exocytosis. Sentinel goblet cells (senGCs), which are located at the colonic crypt entrance, responded to TLR-ligands interaction via Nlrp6 signaling, and this has been reported to trigger calcium ion-dependent compound exocytosis of MUC2 from the senGCs and generate the intercellular gap junction signal to induce MUC2 secretion from adjacent goblet cells (Birchenough et al., 2016). Recently, using a *Vamp8*^–/–^ animal model, Cornick et al. (2019) illustrated that VAMP8 promoted the exocytosis of MUC2 protein from colonic goblet cells to maintain innate intestinal homeostasis, and the absence of VAMP8 caused an altered mucus layer and increased encounters with microbial antigens, resulting in high susceptibility to both chemical and infectious colitis.

MUC2 protein serves as an important component to protect the intestinal tract in the following ways. (1) It induces an anti-inflammatory effect. MUC2 provides substantial assistance in the synthesis of antimicrobial peptides, blocks the induction of inflammation, and protects the intestinal mucosa from the destruction of sodium gluconate (Cobo et al., 2017). The decreased expression of MUC2 in the intestinal cavity leads to the invasion of various pathogenic microorganisms and toxic substances, causing intestinal epithelial cell apoptosis, abnormal distribution, and expression of tight junction proteins, resulting in increased intestinal mucosal permeability and impaired intestinal mucosal barrier function (Xu et al., 2012). A deep targeted resequencing of 122 genes in Dutch ulcerative colitis (UC) patients was performed and identified an association of rare variants in the MUC2 gene with UC in the Dutch population but not in the German population, suggesting a putative, population-specific role of MUC2 in UC susceptibility (Visschedijk et al., 2016). (2) In addition, MUC2 maintains homeostasis of the intestinal environment. Susceptible intestinal bacteria are protected by its the antimicrobial effect of MUC2. *In vitro* and *in vivo* experiments showed that a lack of MUC2 impaired β-defensin mRNA expression and peptide localization in the colon, causing intestinal microecological and intestinal bacteria imbalance, and endotoxin translocation and increasing the occurrence of spontaneous intestinal inflammation (Cobo et al., 2015). The long-term colonization of intestinal microflora is also required to maintain normal intestinal mucus layers (Johansson et al., 2015), indicating that the relationship between the mucus layer and microflora is mutually beneficial. (3) MUC2 stimulates immune function. Mucins are large O-glycoproteins with numerous O-oligosaccharide chains in their skeleton, which not only provide binding sites for immune molecules, such as secretory immunoglobulin A (IgA) and antimicrobial peptide, but also encapsulate harmful substances at the mucosal surface. Finally, with the cooperation of dendritic cells, mucins could induce the expression of interleukin (IL)-10 and other immune factors and then play a supportive role in the immune response (Johansson et al., 2013).

It is accepted that MUC2 is expressed in normal colon epithelial cells, but abnormal levels of MUC2 can be found in various malignant tumors and precancerous lesions. In patients with UC, a precancerous lesion of colon cancer, the expression of MUC2 was decreased, while in *MUC2*^–/–^ mice, the early phase of spontaneous colitis was caused by a defective mucus barrier and subsequent contact of bacteria with the intestinal epithelium (Wenzel et al., 2014). A mass spectrometry analysis of mucus in the colon tissues of UC patients showed that the core mucus structural components, including MUC2, were significantly reduced in active UC, even in noninflamed segments, predicting the attenuation of the goblet cell secretory response to microbial challenge independent of local inflammation (van der Post et al., 2019). However, the expression of MUC2 in colorectal cancer is different based on the histopathological type (Kasprzak et al., 2018). In mucinous adenocarcinoma, the expression of MUC2 is significantly elevated, possibly due to the maintenance of Atoh1 expression by the SCF/c-kit signaling pathway, which leads to mucinous colorectal adenocarcinoma (MCA) (Shen et al., 2018). In the nonmucinous type of colon cancer, decreased MUC2 expression was found, and expression was found to be suppressed by the transcriptional factor caudal type homeobox 2 (CDX2), which may have been caused by the high methylation modification of the promoter of the MUC2 gene (Hanski et al., 1997). However, in CRC cells, a glycosylation defect of the MUC2 gene in cancer cells leads to a failure to express the normal mucus type (Yonezawa and Sato, 1997).

By analyzing the relationship between MUC2 expression and clinicopathological parameters in patients with colorectal cancer, Li et al. (2018) found that MUC2 expression was negatively correlated with TNM stage and lymphatic metastasis, but positively associated with survival. However, in the Finland population, Elzagheid et al. (2013) reported the correlation of MUC2 expression with the location of the tumor, recurrence, and prognosis of CRC. It is interesting that low MUC2 expression showed a preferential relationship with the left-sided colon cancer and indicated a short disease-free survival (DFS) and disease-specific survival (DSS), suggesting that MUC2 may be a potential biomarker for differentiating left/right colon cancer and evaluating the prognosis of patients. Hsu et al. (2017) suggested that low expression of the MUC2 gene was related to metastasis of colorectal cancer and was related to the activation of IL-6 signaling.

In terms of influencing treatment, Leteurtre et al. (2004) investigated the tolerance of the colon cancer cell line HT-29 clone to the chemotherapeutic drug 5-fluorouracil (5-FU), and found that HT29-5F12, a mucus-secreting clone mainly expressing MUC2, was composed of nonpolarized cells secreting mucus with anti-colonic mucin immunoreactive activity, suggesting the valuable role of MUC2 in drug resistance pathways. Another study, reported by the same group, showed that these clones resist chemotherapy by acquiring stemness and quiescence and that these phenotypes were associated with the c-Yes/YAP axis (Touil et al., 2014). According to the conclusion of the study, the following questions come into view. Is the MUC2 gene overexpression in HT29-5F12 cells related to resistance to 5-FU? What is the relationship between overexpression of MUC2 and the c-Yes/Yap axis? It is necessary to study these problems more carefully. In addition, Rotkrua et al. (2011) found that miR-9 may promote the proliferation of MKN45 and NUGC-3 gastric cancer cell lines by inhibiting CDX2 and its downstream targets (such as MUC2), which suggests that the anti-miR-9 drugs can indirectly regulate the expression of MUC2 and delay the progression of gastric cancer, which also has important significance for colorectal cancer.

To date, there are few studies on the regulatory mechanisms of MUC2 expression between mucinous and nonmucinous adenocarcinoma, and limited samples have been studied. Based on the current and updated evidence, an understanding of the potential tumor-suppressive role of MUC2 in CRCs is forthcoming, and more investigations are needed to uncover its underlying mechanism.

## MUC5AC

MUC5AC, encoded by the *MUC5AC* gene on chromosome 11p15.5 (Figure 2B), belongs to gastric type mucins and is mainly expressed in gastric goblet cells (Reis et al., 1999), but is also expressed in the trachea, bronchial mucosa and cervical endometrium (Bartman et al., 1999); it is not found in normal colorectal epithelial cells (Fagerberg et al., 2014). The tandem repeat unit of MUC5AC is separated by many cysteine-rich subdomains (Guyonnet Duperat et al., 1995).

Abnormal expression of MUC5AC can be seen in malignant tumors and precancerous lesions. As described above, there seems to be much in common between the MUC2 gene and the MUC5AC gene, both at the level of sequence homology and in the molecular mechanism controlling the transcription and expression of the MUC gene (Van Seuningen et al., 2001). Byrd et al. reviewed all mucins and mucin-binding proteins in colorectal cancer and suggested that the MUC2 gene and MUC5AC gene were expressed simultaneously in mucus secreting cells during carcinogenesis, which may be due to the common regulatory mechanism, namely, pKa, PKC, PKG, Ca2, and SP1/SP3 signal transduction) (Byrd and Bresalier, 2004). Perrais et al. (2002) investigated the regulation of the 11p15.5 mucin gene promoter and found that in lung cancer cells, MUC2 and MUC5AC are two target genes of the epidermal growth factor receptor (EGFR) ligand and are upregulated due to the activation of the EGFR/Ras/Raf/extracellular signal-regulated kinase-signaling pathway and the recruitment of transcription factor Sp1 to the promoter region of MUC2/MUC5AC, suggesting Sp1 as a regulator of both MUC2 and MUC5AC expression. DNA hypomethylation levels were also involved in the regulation of both MUC2 and MUC5AC expression in CRC and were associated with poor differentiation and microsatellite instability (MSI) status; in particular, MUC5AC hypomethylation was strongly associated with MSI status in cancer, suggesting MUC5AC demethylation as a useful hallmark for MSI in CRC (Renaud et al., 2015). In addition to the HT29-5F12 clone, Leteurtre et al. (2004) also provided a mucus-secreting clone, HT29-5M21, which was composed of monolayered polar cells secreting mucus with strong anti-gastric mucin immunoreactivity, mainly expressing MUC5AC and MUC5B, and showing resistance to MTX and sensitivity to 5-FU, suggesting the different functions and chemotherapy sensitivities of MUCs.

Unlike MUC2, the expression of MUC5AC can be found to different degrees during the development of CRC, specifically both in mucinous and nonmucinous adenocarcinomas. Using siRNA technology, Zhu et al. knockdown the expression of MUC5AC in SW620 cells, a CRC cell line with high MUC5AC expression, and found that siMUC5AC significantly inhibited cell migration and invasion and induces cell apoptosis and G1-phase cell cycle arrest; these effects resulted in impaired colony formation (Zhu et al., 2016), suggesting that MUC5AC is a promising target for the treatment of colon cancer. Unsurprisingly, CRC patients with high MUC5 expression showed poor cell differentiation, a high lymph node metastasis rate and late stage CRC (Wang et al., 2017), which suggests that MUC5AC is a promoter of tumors. However, some studies believe that MUC5AC is a protective factor for patients with CRC. For example, Imai et al. (2013) reported that expression of MUC5AC in CRC decreases with increasing malignancy, and the loss of MUC5AC expression may be a prognostic factor for aggressive colorectal adenocarcinoma, which suggests that patients with high expression of MUC5A have a better prognosis. Betge et al. (2016) confirmed that CRC patients with a high level of MUC5AC do have a longer progression-free survival (PFS) than those with a low level of MUC5AC, especially stage II and III CRC patients. The controversial results need further investigation to explore the role of MUC5AC and the underlying molecular mechanisms in patients with CRC.

As the expression of MUC5AC in colorectal cancer tissues is abnormal, what is the humoral immune response to this abnormal protein expression? Will it cause an immune response *in vivo* to affect normal tissues? Kocer et al. (2006) measured free circulating MUC5AC antibodies and confirmed that serum anti-MUC5AC antibody was detected in 27.3% of healthy people (6 of 22), 45% of polyp patients (9/20) and 60% of CRC patients (18/30). Importantly, CRC patients with high serum anti-MUC5AC antibody positivity were predicted to have advanced stage disease, and poorly differentiated tumors especially showed poor prognostic parameters, DFS and overall survival (OS); these factors may be associated with the decreased expression of the MUC5AC gene in tumor tissues. The findings provide novel therapeutic strategies for targeting the MUC5AC gene in tumor tissues or the anti-MUC5AC antibody in serum, benefiting potential CRC patients with precision medicine.

## MUC5B

The total length of the MUC5B gene is 39.09 kb with 48 exons, encoding a 5,662 amino-acid peptide (Desseyn et al., 1998) that contains one Sp1 binding site (NAU62) through which specific interactions with nuclear factors affecting its transcriptional regulation can occur (Pigny et al., 1996b; Figure 2C). Unsurprisingly, consistent with the regulatory mechanism of MUC2 and MUC5AC, hypermethylation of the MUC5B promoter is also the main mechanism of its silencing (Vincent et al., 2007).

MUC5B is mainly expressed in the bronchus, submandibular gland, cervix, pancreas, and gallbladder and is the main component that maintains lubrication and viscoelasticity of saliva, normal lung mucus and cervical mucus (Vandenhaute et al., 1997; van Klinken et al., 1998; Fagerberg et al., 2014). Van Seuningen et al. (2000) investigated the 5′-flanking region and promoter activity of MUC5B in colon cancer cell lines and showed a cell-specific manner of *MUC5B* promoter activities, as *MUC5B* it is very active in mucus-secreting LS174T cells, whereas it is inactive in Cac-2 enterocytes and HT-29 undifferentiated cells. MUC5B upregulation has been found in human diseases, such as sinus mucosa polyps (Wu et al., 2011), nasal polyps (Amini et al., 2019), chronic obstructive pulmonary disease (Hancock et al., 2018), and Helicobacter pylori-related gastropathy (Zhang et al., 2018), suggesting the important role of MUC5B and its involvement in the pathogenesis of these diseases. In gastric and intestinal cancer cell lines, the expression of MUC5B was also found to be increased, such as HT-29 MTX cells (Lesuffleur et al., 1995) and LS174T cells (van Klinken et al., 1998). Jiang et al. (2015) demonstrated that the variant rs35705950 in the promoter of the MUC5B gene region was significantly associated with increased susceptibility to idiopathic pulmonary fibrosis (IPF), increased severity of disease and poor OS of patients. Dressen et al. (2018) conducted whole-genome sequencing in people of European ancestry to assess telomere length and identify rare altered protein variants encoded by the rs35705950 risk allele, suggesting that multiple genetic factors contribute to sporadic idiopathic pulmonary fibrosis (IPF) associated with MUC5B.

Further studies investigated the effect of this abnormal expression of the MUC5B gene on the biological behavior of different types of cancers. In lung carcinoma, the MUC5B gene was illustrated to be upregulated by the long non-coding RNA MUC5B-AS1, promoting lung cancer cell mobility *in vitro* and metastasis *in vivo*, which was associated with poor outcomes in patients with lung carcinoma (Yuan et al., 2018). A regulatory linkage between dual-specificity phosphatase 28 (DUSP28) and MUC5B/MUC16 was reported in pancreatic cancer cells by a study to elucidate the underlying mechanism by which DUSP28 promotes the development of pancreatic cancer (Lee et al., 2016). The overexpression of the MUC5B gene was reported to lead to the aggressive behavior of breast cancer cells (Valque et al., 2012), and silencing the MUC5B gene efficiently recovered the sensitivity of breast cancer cells to chemotherapeutic drugs by impairing the maturation of dendritic cells and inducing an antitumor immune response (Garcia et al., 2016).

However, research focused on the MUC5B gene in colorectal carcinomas is limited. In the intestinal cancer cell line LS180, the proinflammatory cytokines IL-6 triggered the expression of MUC2, MUC5B, and MUC6 genes, and promoted their secretion, whereas IL-1 or tumor necrosis factor-α (TNF-α) activated the expression of MUC2 and MUC5AC genes, and alteration of mucus layers was induced by these differentially expressed cytokines (Enss et al., 2000). Based on genome-wide association studies (GWAS) in the Swedish population, rs200554635 in the MUC5B gene was reported by Jiao et al. (2018) which is predicted to affect carcinogenesis and clinical outcomes in CRC patients. To understand the effect of abnormal expression of the MUC5B gene on the malignant biological behavior of cancer cells, Lahdaoui et al. (2017) knockdown the expression of the MUC5B gene in the human gastric cancer cell line KATO-III and the colon cancer cell line LS174T and found that the downregulation of the MUC5B gene induced a decrease in cell proliferation and migration *in vitro* and *in vivo*, partially mediated by the Wnt/β-catenin pathway. In addition, Garcia et al. (2016) used short hairpin RNA (shRNA) to knockdown the expression of the MUC5B gene in the breast cancer cell line MCF-7. The results showed that reducing the expression of the MUC5B gene could inhibit the cell adhesion, cell growth and clonogenic ability of MCF-7cells but did not increase apoptosis (Reynolds et al., 2019). Valque et al. (2012) highlighted how MUC5B leads to aggressive behavior in MCF7 cells, and they showed that MUC5B promoted proliferation and invasion *in vitro* and enhanced growth and cell dissemination *in vivo*. The abnormal expression of the MUC5B gene can also affect the resistance of cancer cells to chemotherapy drugs. Garcia et al. (2016) also showed that MUC5B was associated with a worse response to chemotherapy and that reducing the expression of MUC5B increased chemosensitivity (Reynolds et al., 2019). Leteurtre et al. (2004) found that HT29-5M12 cells, which mainly express the MUC5B gene, were resistant to chemotherapeutic drugs such as MTX and 5-FU. Therefore, assessment of drug sensitivity in CRC patients with high expression of MUC5B and resistance to MTX or 5-FU for follow-up chemotherapy will greatly improve the effectiveness of treatment and compliance of patients. Of course, this mechanism is not very clear at present, and it needs to be verified in other types of CRC cell lines and *in vivo* experiments.

## MUC6

In 1993, the MUC6 gene was first named and reported in a gastric mucosal cDNA library by Toribara et al. (1993). The cDNA of the MUC6 gene is characterized by a tandem repeat region with a 507 bp-long individual repeat unit and the protein sequence of the MUC6 gene is rich in threonine, serine, and proline, containing a relatively large amount of histidine and alanine (Toribara et al., 1993). The N-terminal organization of the MUC6 gene is highly similar to that of the MUC2 gene, both of which contain vWF D-like domain (Rousseau et al., 2004). Although MUC6, similar to MUC5AC, belongs to gastric mucins, it is mainly expressed in glandular epithelial cells, as well as in the gallbladder, pancreas, and duodenum (Reis et al., 1999; Fagerberg et al., 2014; Figure 2D).

Investigations into the regulatory mechanism of MUC6 gene expression have mainly focused on gastrointestinal diseases. Hath1, an important transcription factor in the Notch signaling pathway, was found to transcriptionally increase the mRNA levels of the MUC6 and MUC5AC genes in gastric cancer cell lines, suggesting the potential role of Hath1 in the development of gastric cancer through transcriptional regulation of the MUC6 and MUC5AC genes (Sekine et al., 2006). The promoter region of the MUC6 gene contains a TATA box at −35 bp to −29 bp, an NF-κB consensus sequence at −173 bp to −164 bp, and Sp. family binding sites at −530 bp to −521 bp and −847 bp to −838 bp. Sakai et al. (2005) conducted luciferase assays and confirmed that NF-κB and Sp. family members are transcriptional factors that regulate the expression of MUC6.

On the other hand, the abnormal expression of the MUC6 gene, usually associated with increased tumor cell mobility, has been found in many malignant tumors, such as gastric cancer (Taniyama and Taniyama, 2017), duodenal cancer (Toba et al., 2018), breast cancer (Rakha et al., 2005), pancreatic cancer (Ohya et al., 2017), endometrial cancer (Hodgson et al., 2019), CRC (Tsai et al., 2015), and lung cancer (Duruisseaux et al., 2017). In a case-control study of gastric cancer, five minisatellites (MS1-MS5) were identified in the genomic structure of MUC6, and MUC6-MS5 alleles from cancer-free controls and individuals with gastric cancers were scored. The results showed that the increased incidence of short rare MUC6-MS5 alleles was statistically significant in gastric cancer patients compared to age- and sex-matched cancer-free controls (Kwon et al., 2010). Interestingly, upregulation of MUC6 expression was found in the early stage and absent in the late stage of pancreatic cancer (Leir and Harris, 2011). Overexpression of MUC6 glycoprotein domains significantly inhibited tumor cell adhesion to matrix proteins in LS180 cells (not found in PANC-1 cells), and the N- and C-terminal domains of MUC6 inhibited invasion of both LS180 and PANC-1 cells, suggesting that the MUC6 gene may prevent tumor cells migrations through the basement membrane of the pancreatic duct and suppress the development of infiltrating carcinoma (Leir and Harris, 2011).

However, whether the MUC6 gene can be used as a useful biomarker for the progression of colorectal cancer remains to be further studied. Positive expression of the MUC6 gene was found in colonic hyperplastic polyps and serrated adenomas, and positive expression of the MUC6 gene was also identified in crypt cells (Gibson et al., 2011). However, Gibson et al. (2011) indicated that it was unreliable to use the expression of the MUC6 gene to distinguish proliferative polyps from sessile serrated adenomas/polyps or sessile serrated adenomas/polyps with dysplasia because of the lack of specificity. They also demonstrated that although polyps from the left and right hemi-colons showed positive expression of the MUC6 gene, the positive rate of MUC6 expression in the right hemi-colons of sessile serrated adenoma and traditional serrated adenoma was significantly higher than that in the left hemi-colons of sessile serrated adenoma and traditional serrated adenoma, maybe due to the biological differences among adenomas in different parts of the colon involving the malignant transformation pathway of serrated adenomas (Gibson et al., 2011). Therefore, the relationship between MUC6 and sessile serrated adenoma is controversial. Bartley et al. (2010) found that the expression of the MUC6 gene was strongly associated with the proximal location of serrated polyps but only provided modest utility as a tissue biomarker for sessile serrated adenoma due to relatively low sensitivity. Conversely, the coexpression of MUC5AC and MUC6 genes can be detected in proliferative polyps of the colon with gastric metaplasia, but the specific expression mechanism is still unclear. Betge et al. (2016) examined the expression of the MUC6 gene in 381 CRC tissues by immunohistochemistry and analyzed the survival information of CRC patients. They found that patients with deficient expression of the MUC6 gene showed short PFS, while patients with MUC6 overexpression had long PFS and cancer-specific survival (CSS), especially in stage II and III CRC, indicating the protective role of the MUC6 gene in the occurrence and development of CRC, which was related to favorable outcomes of CRC patients (Betge et al., 2016).

## Discussion and Perspectives

Mucins are part of a high-molecular-weight epithelial glycoproteins family and have clustered oligosaccharides linked to tandem repeat peptides through O-glycosidic linkages. Among them, the secreted gel-forming mucins MUC2, MUC5AC, MUC5B, and MUC6 are structurally and functionally distinguished from the transmembrane mucins and are encoded by genes located in a similar region, chromosome 11p15.5. Unsurprisingly, there are many similarities in gene regulation and function. Usually, two or more MUCs are coexpressed during the development of many diseases, suggesting the detection of MUC expression as an important tool for clinical diagnosis, treatment and prognosis analysis. The current findings of the expression and function of the MUC gene cluster located on chromosome 11p15.5 in CRC were systematically reviewed above.

In CRC, the activation of the promoter CpG island methylation phenotype, MSI and multiple signaling pathways has been proven to be involved in the regulation of mucin differentiation. For example, specific DNA methylation of the promoter of the MUC2 gene has been illustrated to regulate its transcription. Hypomethylation of the promoter of the MUC5AC gene is significantly related to its protein expression level, poor differentiation, and MSI. Hypermethylation of the promoter of the MUC5B gene is the major mechanism responsible for its silencing, while methylation of the promoter of MUC6 gene is not related to its silencing (Vincent et al., 2007).

Moreover, transcription factors also play different roles in the regulation of MUC gene expression. For example, the Notch/Hath1 axis transcriptionally regulates the expression of MUC6 and MUC5AC genes (Li et al., 2018), while the transcription factors Sp1, SOX9, and CDX2 can participate in the transcriptional regulation of many MUC genes, including MUC2 (Sakai et al., 2005). Furthermore, mutations of tumor suppressor genes, such as p53 and RAS, are involved in the regulation of MUC genes located on chromosome 11p15.5 (Ookawa et al., 2002). Finally, different signaling pathways, such as the EGFR-RAS-RAF signaling pathway, Wnt signaling pathway, and Notch signaling pathway, have also been reported to be involved in the regulation of MUC gene expression.

As the main component of the mucus layer, the secretory mucin MUC2 plays an important role in protecting the intestinal tract through the involvement of the anti-inflammatory response, maintenance of intestinal environment homeostasis and activation of immunity. Although the role of MUC2 in CRC is supposed to be tumor suppressive, the expression of the MUC2 gene or other MUC genes in CRC is controversial in different studies (Wang et al., 2017). First, MUC2 is mainly expressed in normal colon epithelial cells, and there is a difference in MUC2 expression between mucinous carcinomas and nonmucinous carcinomas, suggesting the diverse molecular mechanisms involved in these processes (Walsh et al., 2013). The gastric mucus, MUC5AC is mainly expressed in gastric goblet cells but not in normal colorectal epithelial cells. During the development of CRC, abnormal expression of the MUC5AC gene was found in different stages of CRC, and MUC5AC was expressed in both mucinous and nonmucinous adenocarcinoma (Kocer et al., 2006). MUC5B is the main component that keeps saliva, normal lung mucus and cervical mucus lubricated and viscoelastic. Overexpression of MUC5B gene has been found in some subtypes of gastric and intestinal cancer, but the molecular mechanisms remain unknown (Lahdaoui et al., 2017). Another gastric mucus, MUC6 is mainly expressed in glandular epithelial cells and is absent in many tumors (Betge et al., 2016), predicted to have a tumor-suppressive role in the occurrence and development of CRC.

Correlation analysis of MUC expression and clinicopathological parameters of patients with CRC found that MUC2 and MUC5AC were correlated with tumor stage, lymph node metastasis and prognosis of patients, and could be used as biomarkers to evaluate the prognosis of patients (Li et al., 2018), while the overexpression of MUC5B in CRC cells seemed to be related to degree of malignancy (Walsh et al., 2013), and increased expression of MUC6 was associated with malignancy and poor prognosis in CRC cells (Betge et al., 2016). Based on the current findings, the detection of MUC expression in CRC patients will benefit patients in the following aspects. (1) For diagnosis, MUCs on chromosome 11p15.5 are new potential biomarkers for CRC patients. For example, the differential expression of MUC2 in mucinous/nonmucinous adenocarcinoma, and left/right colon cancer may be an important clue for differential diagnosis. Positivity for anti-MUC5AC antibody in plasma may become a favorable indicator for early screening of CRC. (2) For treatment, MUCs may function as new therapeutic targets for CRC patients. In addition, the potential roles of MUCs in the signaling pathways involved in drug resistance can guide follow-up and in-depth studies and optimize treatment options for patients with CRC in the selection of chemotherapeutic drugs to improve the curative effect, save medical costs and improve prognosis. (3) For predicting prognosis, the advantages of MUCs, especially MUC2 and MUC5AC, are obvious in the current findings. CRC patients without MUC2 expression had short DFS and DSS, predicting poor prognosis. Although the relationship between MUC5AC and the survival of CRC patients is controversial, the detection of MUC5AC is also beneficial for predicting prognosis in combination with other factors. As a potential tumor suppressor, the overexpression of MUC6 is related to favorable outcomes in CRC patients. The current findings indicated the protective roles of MUC2 and MUC6 in the occurrence and development of CRC, while MUC5B was identified as a pathogenic factor. For the controversial role of MUC5AC, further investigation needs to be performed. In the future, more large-scale multicenter experiments are needed to further study the differences in regulatory mechanisms at the molecular level, which is of great significance to further understand the mechanism of CRC and to find new targeted drugs.

## Conclusion

In conclusion, most of the studies on the expression and roles of the MUC gene cluster located on chromosome 11p15.5 in CRC are confined to the functional level; comprehensive analysis is limited, and research involving molecular and genetic levels is still lacking. The epigenetic and transcriptional regulatory mechanisms of the MUC gene clusters located on chromosome 11p15.5 deserve to be further explored, and the relationships and differences between these genes deserve more attention, especially in the case of coexpression or differential expression of MUCs in CRC. Therefore, a large number of in-depth, multidimensional and high-quality studies are needed to reveal the roles of MUCs in CRC. It is believed that soon, the roles of MUC genes in the diagnosis, treatment and prognostic evaluation of CRC patients will provide insights and hope t patients and clinicians.

## Author Contributions

H-TW and WL contributed to the conception and design of the study and critically revised the original manuscript. G-LG and JL organized the database, searched the literature, and structured and carefully drafted the manuscript. W-JC, Q-QY, and YX analyzed and interpreted the data, drafted and revised carefully the manuscript. All authors contributed to manuscript revision, read, and approved the submitted version.

## Conflict of Interest

The authors declare that the research was conducted in the absence of any commercial or financial relationships that could be construed as a potential conflict of interest.
